# Multiscale Distribution Entropy Analysis of Short-Term Heart Rate Variability

**DOI:** 10.3390/e20120952

**Published:** 2018-12-11

**Authors:** Dae-Young Lee, Young-Seok Choi

**Affiliations:** Department of Electronics and Communications Engineering, Kwangwoon University, Seoul 01897, Korea

**Keywords:** electrocardiogram, heart rate variability, multiscale distribution entropy, RR interval, short-term inter-beat interval

## Abstract

Electrocardiogram (ECG) signal has been commonly used to analyze the complexity of heart rate variability (HRV). For this, various entropy methods have been considerably of interest. The multiscale entropy (MSE) method, which makes use of the sample entropy (SampEn) calculation of coarse-grained time series, has attracted attention for analysis of HRV. However, the SampEn computation may fail to be defined when the length of a time series is not enough long. Recently, distribution entropy (DistEn) with improved stability for a short-term time series has been proposed. Here, we propose a novel multiscale DistEn (MDE) for analysis of the complexity of short-term HRV by utilizing a moving-averaging multiscale process and the DistEn computation of each moving-averaged time series. Thus, it provides an improved stability of entropy evaluation for short-term HRV extracted from ECG. To verify the performance of MDE, we employ the analysis of synthetic signals and confirm the superiority of MDE over MSE. Then, we evaluate the complexity of short-term HRV extracted from ECG signals of congestive heart failure (CHF) patients and healthy subjects. The experimental results exhibit that MDE is capable of quantifying the decreased complexity of HRV with aging and CHF disease with short-term HRV time series.

## 1. Introduction

An electrocardiogram (ECG) is a record of electrical activity caused by the heart. ECG is a non-invasive tool that is effective for a variety of biomedical applications such as heart rate measurement, diagnosis of heart failure, emotion recognition, and so on [1]. One of the main areas of need for ECG analysis is the diagnosis of heart diseases. Since ECG is closely related to cardiac activity, it can play an important role in the diagnosis of heart diseases. Among the causes of many heart diseases, congestive heart failure (CHF) is a collective term for heart disease that causes congestion in the systemic venous system due to heart pumping dysfunction [2]. Since heart failure diseases may cause death to many people all over the world every year, the diagnosis of CHF is of great interest and remains challenging issue.

Among the features that can be extracted from the ECG recordings, variabilities in heart beat-to-beat intervals controlled by the autonomic nervous system (ANS) is usually used, which is referred to as heart rate variability (HRV) [3]. The HRV analysis helps us to represent CHF symptoms and is widely used to identify CHF patients [4]. In practice, the HRV analysis using short-term inter-beat (RR) interval is of greatly important because of its suitability for short-term patient monitoring and the need for almost immediate reception of test results. Therefore, the complexity analysis of HRV is mainly utilized for the distinction between healthy people and those with heart disease, such as CHF patients. Recently, it is known that HRV of a healthy person exhibits dynamic fluctuations and it is characterized by a decrease in the incidence of CHF heart disease and aging [5,6].

Quantitative analysis of the complexity of a time series is promising in analyzing physical, mechanical, and biological systems that exhibit non-static, nonlinear, and complex behaviors [7,8]. A quantitative measure of physiological signal plays an important role for computer-aided diagnosis in clinical applications [9]. In this regard, various entropy approaches have attracted attention in the complexity analysis [10]. Conventional entropy measures such as sample entropy (SampEn) [11], fuzzy entropy (FuzzyEn) [12], and permutation entropy (PE) [13] have been utilized for the complexity analysis of HRV [14,15,16,17]. However, since these methods measure the irregularity of the time series, the resultant quantifications may fail to characterize the complexity of the underlying time series. For example, the SampEn value of white Gaussian noise is assigned to be higher than that of 1/*f* noise, which is not consistent with the complexity analysis in the sense that 1/*f* noise has higher complexity owing to its long-range correlations [18]. Along this line, though the complexity of HRV of healthy person is higher compared to that of patients, the conventional entropy approaches may fail to reflect the higher complexity of HRV of healthy over diseased status.

To address this issue, Costa et al. [5,19] have proposed a multiscale entropy (MSE) method that consists of a coarse-graining process and SampEn computation to measure the complexity of a time series at different temporal scales. It is generally effective in identifying characteristics over multiple temporal scales because the biological system possesses distinct properties over several spatial and temporal scales [19]. Therefore, various studies using this MSE method have been performed to analyze the complexity of HRV on various temporal scales [20,21]. Subsequently, the coarse-graining process has been applied to the FuzzEn and PE methods, yielding the multiscale fuzzy entropy (MFE) [22] and multiscale permutation entropy (MPE) [23], respectively. However, the coarse-graining process reduces the length of the coarse-grained time series as scale increases, thus resulting in inaccurate or undefined entropy computation. This behavior of MSE makes it unsuitable for computing entropy of a short-term time series. Wu et al. [24] have proposed a modified multiscale procedure that uses a moving-averaging process instead of a coarse-graining process. The authors have shown that the use of a moving-averaging process leads to better capability to reflect long-range correlations of a short-term time series than a coarse-graining one. Thus, it can provide more reliable computation of entropy values in situations in which a short-term time series is given.

Moreover, the MSE and MFE methods have drawbacks of high dependency on predetermined parameters because they do not fully make use of the distance information between vectors in the state space during computation. Recently, the distribution entropy (DistEn) proposed by Li et al. [25] has been developed from the fact that the inherent information of the distances between vectors in the state space is maximized through the probability density estimation, leading to relatively lower sensitivity to predetermined parameters and data length. While SampEn makes use of uses only a fraction of the distance vector information, DistEn is capable of quantifying full distance information. It gives DistEn improved sensitivity and consistency. However, DistEn only considers the complexity computation at single scale. 

Here, we proposed an effective way to quantify HRV using the short-term RR interval of ECG signals. The proposed method is based on a computation of DistEn over multiple scales by a moving-averaging process, which is referred to as the multiscale distribution entropy (MDE). The computation of the MDE, which inherits the merits of the DistEn, is able to address the shortcoming of the conventional MSE which may fail to capture the long-range correlation of the short-term time series. We compare the performance of MDE to the conventional MSE using several synthetic data by evaluating the stability and characteristics over multiple temporal scales for the short-term time-series. Then, the capability of the proposed MDE is examined for the RR intervals with various lengths extracted from actual ECG signals of the healthy subjects and the CHF patients. 

The remainder of this paper is organized as follows: In Section 2, we describe the conventional entropies and the proposed MDE. In Section 3, the results on synthetic data and real ECG data are presented. Section 4 presents the conclusions of this work.

## 2. Materials and Methods 

### 2.1. Sample Entropy

The SampEn method is a modified entropy computation from the approximate entropy (ApEn) method [8]. SampEn computes the conditional probability that quantifies that the similarity of two sequences of different length m and m+1 is maintained. Here, m denotes the length of sequences that are compared to each other. More specifically, the SampEn method consists of four steps: reconstruction, definition of distance, definition of the criterion for similarity, and entropy calculation. 

First, for a *N* points time series $x_{N}=\left\{x_{1},\;x_{2},\;\ldots ,\;x_{N}\right\}$, it is to reconstruct $x_{N}$ into multidimensional vectors as follows:

(1) $$X_{m}^{\tau }\left(i\right)=\left\{x_{i},\;x_{i+\tau },\;\ldots ,\;x_{i+\left(m-1\right)\tau }\right\}$$

where m denotes the embedding dimension and τ denotes the time delay factor. 

Next, define the distances between two different vectors as the maximum difference of their corresponding components as follows:

(2) $$d\left[X_{m}^{\tau }\left(i\right),X_{m}^{\tau }\left(j\right)\right]=\max\left\{\left|x_{i+k\tau }-x_{j+k\tau }\right|:0\leq k\leq m-1\right\}$$

where *i* and *j* are not equal and the distance $d\left[X_{m}^{\tau }\left(i\right),X_{m}^{\tau }\left(j\right)\right]$ is referred to as the Chebyshev distance.

Third, if the distance $d\left[X_{m}^{\tau }\left(i\right),X_{m}^{\tau }\left(j\right)\right]$ is less than a threshold parameter r, a match occurs and we count the number of vector pairs that satisfy this condition. This process proceeds when the embedding dimension is *m* and m+1, which are called $B_{i}^{m}$ and $B_{i}^{m+1}$, respectively.

(3) $$B^{m}=\frac{1}{N-m\tau }\sum_{i=1}^{N-m\tau }B_{i}^{m},\;B^{m+1}=\frac{1}{N-m\tau }\sum_{i=1}^{N-m\tau }B_{i}^{m+1}$$

Finally, SampEn is defined by

(4) $$\mathrm{SampEn}\left(x_{N},m,r,\tau \right)=-\ln\left[\frac{B^{m+1}}{B^{m}}\right]$$

In general, r is selected in the range of [0.1σ,0.25σ], where σ is the standard deviation of original time series $x_{N}$ [11]. 

### 2.2. Distribution Entropy

The DistEn method quantifies the amount of information in the state space of the univariate time series by estimating the distribution characteristic of the distances between vectors [25]. The computation of DistEn consists of four steps: reconstruction, construction of a distance matrix, probability density estimation, and entropy calculation.

First, for N points of a time series $x_{n}$, we reconstruct multidimensional vector $X_{m}^{\tau }\left(i\right)=\left\{x_{i},\;x_{i+\tau },\;\ldots ,\;x_{i+\left(m-1\right)\tau }\right\}$, where m is the embedding dimension.

Next, construct the distance matrix $D=\left\{d\left[X_{m}^{\tau }\left(i\right),X_{m}^{\tau }\left(j\right)\right]\right\}$, where the $d\left[X_{m}^{\tau }\left(i\right),X_{m}^{\tau }\left(j\right)\right]$ is the Chebyshev distance.

Third, the distribution characteristics of $d\left[X_{m}^{\tau }\left(i\right),X_{m}^{\tau }\left(j\right)\right]$ should completely quantify the information reflecting the distances matrix D. To do this, we estimate the empirical probability density function (ePDF) of the matrix D by using the histogram approach. Since i and j are different, the diagonal components of the matrix D are excluded. In addition, since the $d\left[X_{m}^{\tau }\left(i\right),X_{m}^{\tau }\left(j\right)\right]$ and the $d\left[X_{m}^{\tau }\left(j\right),X_{m}^{\tau }\left(i\right)\right]$ are the same, only the upper or lower triangular part of the matrix D needs to be considered. If the histogram has B bins, the probability $p_{t}$ of each bin is obtained, where t=1,2,…,B. The value of B is usually selected as an integer value in a range of [512, 1024].

Finally, DistEn is calculated as

(5) $$\mathrm{DistEn}\left(x_{N},m,B,\tau \right)=-\frac{1}{\log_{2}B}\overset{B}{\underset{t=1}{\sum }}p_{t}\log_{2}\left(p_{t}\right)$$

**Figure 1:**
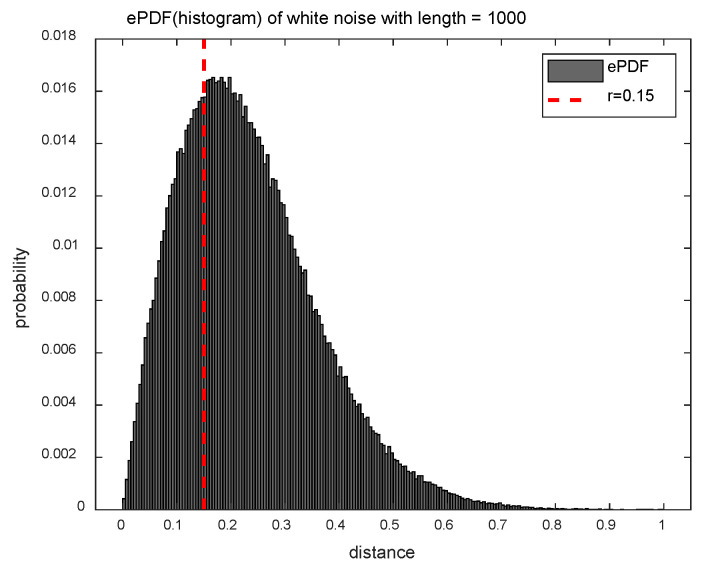
Empirical probability density function (ePDF) of white noise with length N=1000 (m=2, r=0.15σ).

Figure 1 shows the ePDF of the distance matrix D for the white noise (N=1000) for exploiting the difference between the SampEn and DistEn methods in terms of distance information. The SampEn method uses only a fraction of the distance information (less than the threshold parameter r), and it corresponds to the left area of the red dotted line in Figure 1. On the other hand, since the DistEn method takes full advantage of the distance information, it is able of reflecting the complexity that the SampEn method can’t measure.

### 2.3. Multiscale Distribution Entropy

**Figure 2:**
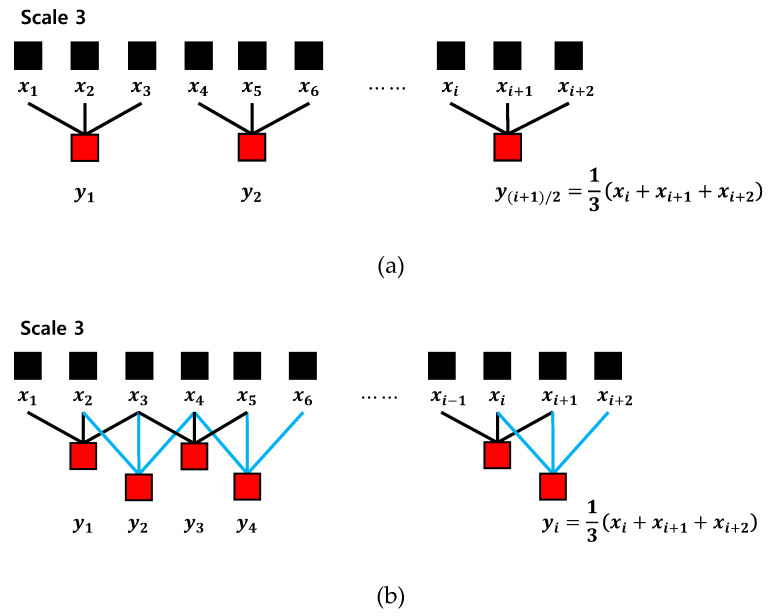
Progress of two multiscale processes: (**a**) coarse-graining; (**b**) moving-averaging.

The coarse-graining procedure generates a number of sets of time series on a time scale s by considering different starting points of the time series. Therefore, the coarse-graining multiscale process can lead to inaccurate entropy values by reducing the length of the time series. To alleviate this drawback, we used the moving-averaging multiscale process, which has a better effect on short-term time series analysis [24]. Figure 2 shows the progress of two multiscale processes. It can be seen that the moving-averaging process (Figure 2b) leads to longer multiscale processed time series compared to the coarse-graining process (Figure 2a). 

The moving-averaging multiscale process is composed of two procedures. First, for a N point time series $x_{N}$ and a given scale factor s, we divide the original time series into several smaller time series overlapped of length scale factor s. Then, the continuous moving-averaged time series are constructed by averaging the number of data points on the scale s as follow:

(6) $$y_{i}^{s}=\frac{1}{s}\overset{i+s-1}{\underset{j=i}{\sum }}x_{j},\;1\leq i\leq N-s+1$$

The moving-averaging process generates multiple sets of time series on the time scale factor s. At the scale factor s=1, the moving-averaged time series $y^{s}$ is equal to the original time series. 

Second, set the time delay factor τ of the DistEn to the scale factor s, and calculate the entropy value of MDE. In other words, the moving-averaged time series on each scale is used as an input signal for entropy calculation of DistEn as follows:

(7) $$\mathrm{MDE}\left(x_{N},m,B,s\right)=\mathrm{DistEn}\left(y^{s},m,B,\;\tau =s\right)$$

### 2.4. Evaluation Data

#### 2.4.1. Synthetic Data

To verify the performance of the MSE and MDE methods with respect to the length of data, we first employed entropy calculation on synthetic data. The synthetic data used in this work are the chaotic series, white Gaussian noise (or simply white noise), periodic signals, and MIX(p) processes. The chaotic series and periodic series are generated from the Logistic attractor x(n+1)=ω×x(n)×(1−x(n)) with ω=4.0 and ω=3.5, respectively. The MIX process is a kind of stochastic signal that is superimposed on a deterministic component, and randomly selected points of N×p are replaced by independent identically distributed random noise in a sinusoidal signal of length N [26]. Finally, white noise is that the values at any pair of times are identically distributed and statistically independent, and it is the case of uncorrelated noise. 

For each signal, 100 realizations were randomly generated with data length of N=100, 300, and 1000, and used for the evaluation of the MSE and MDE.

#### 2.4.2. Real ECG Data

Two real ECG datasets in *PhysioNet* are used [27]. Dataset I includes ECG data from *Fantasia* and *BIDMC CHF*. In addition, Dataset II includes other *CHF RR Interval* and *Normal Sinus Rhythm RR Interval* data. *BIDMC CHF* data includes ECG records from 15 patients with CHF (NYHA classes III, IV) consisting of 11 men aged 22–71 years and 4 women aged 54–63 years. Each record was measured for approximately 20 hours and contains two ECG signals sampled at 250 Hz. *Fantasia* data were measured from 20 healthy people aged 21–34 years and 20 elderly people aged 68–85 years. Each record was measured for approximately 2 hours, and the sampling frequency was 250 Hz. *CHF RR Interval* consists of beat annotation for 29 ECG signals (sampled at 128 Hz) of CHF (NYHA classes I, II, III) patients aged 34–79. In addition, *Normal Sinus Rhythm RR Interval* data consists of beat annotation for 54 ECG signals (sampled at 128 Hz) of subjects in normal sinus rhythm (NSR). 

**Figure 3:**
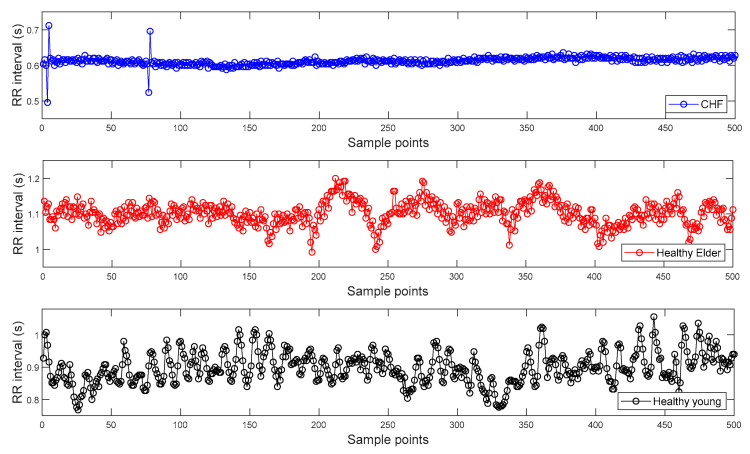
Examples of inter-beat (RR) interval time series extracted from the electrocardiogram (ECG) signals of Dataset I: Congestive heart failure (CHF) patient (**top panel**); Healthy elderly subject (**middle panel**); Healthy young subject (**bottom panel**).

To find the R peak points from the ECG signals of Dataset I, we used a Pan–Tompkins algorithm [28]. Then, RR interval time series are constructed from the distances between two consecutive R peak points and can be seen in Figure 3. Figure 3 shows the representative RR intervals of CHF patient, healthy elderly, and healthy young subjects, respectively.

In this work, we used the RR interval time series of lengths of 100, 300, and 1000 extracted from ECG signals, respectively. Each time series was used for evaluation of the MSE, MPE, and MDE. Firstly, CHF patients (*BIDMC CHF*), healthy elderly, and healthy young groups’ data (*Fantasia*) were analyzed. We then analyzed other CHF patients (*CHF RR Interval*) and NSR subject data (*NSR RR Interval*) to further evaluate the performance of discrimination between CHF patients and normal subjects. The parameters of the MSE and MPE were set to r=0.2σ and m=2, and m=4 and t=1, respectively. The parameters of the MDE were set to m=2 and B=512. 

## 3. Results

### 3.1. Simulation Result Using Synthetic Data

**Figure 4:**
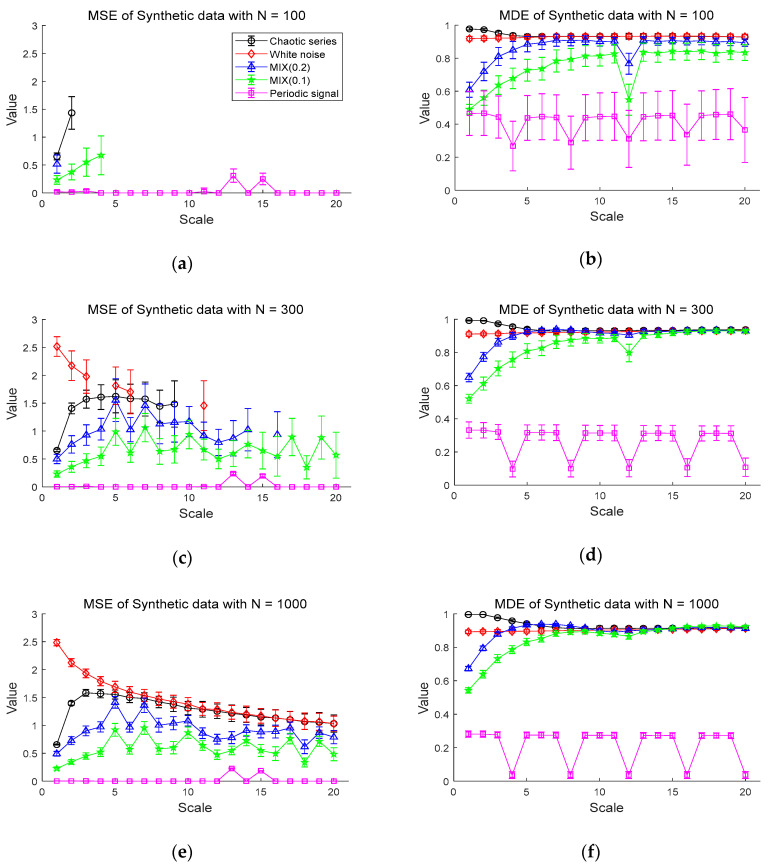
Entropy values for synthetic data: (**a**) multiscale entropy (MSE) value for *N* = 100; (**b**) multiscale distribution entropy (MDE) value for N=100; (**c**) MSE value for N=300; (**d**) MDE value for N=300; (**e**) MSE value for N=1000; (**f**) MDE value for N=1000. Scales between 1 and 20 are used and the value at each scale represents a mean ± standard deviation.

The results of the MDE and MSE evaluation of synthetic data are shown in Figure 4. Figure 4a,b shows the entropy values of the MSE and MDE for time series of length N=100, respectively. Figure 4a shows that chaotic series has the highest values of MSE in the scales less than 3 and the periodic signal has entropy values of almost zero. Notably, the MSE values are mostly only defined on small scales. For instance, the white noise and the chaotic series are defined only on scale 1 and 2, with a large standard deviation. Compared with the MSE results, in Figure 4b, it is evident that the entropy values of MDE become smaller in order of chaotic series, Gaussian white noise, MIX (0.2), MIX (0.1), and periodic series. As the scale increases, the entropy values of MDE for white noise and chaotic series are comparable and higher than those of other synthetic data. The MDE values of MIX (0.1) and MIX (0.2) rise as scale factor increases and remain constant, while the periodic signal keeps the lowest entropy values. In the case of the periodic signal, due to the use of a period of 4 samples, lowest entropy values are repeated on scales of the multiples of 4. 

Next, Figure 4c,d shows the MSE and MDE results for time series of length N=300, respectively. In Figure 4c, white noise has the highest MSE values in a range of small scales. In the range of small scales, the entropy values of chaotic series, MIX (0.2), MIX (0.1), and periodic series follow in order. However, the MSE values of those synthetic data are still undefined over large scale factors, especially for white noise and chaotic series. As can be seen in Figure 4d, the similar results of Figure 4b are observed in Figure 4d in the sense that MDE computation leads to same order of entropy values on small scale factors and stable evaluation over all scales. In addition, it exhibits that the signals, except for the periodic signal, converge to almost similar entropy values on large scales, unlike the result of the data length N=100 in Figure 4b.

Figure 4e,f shows the results of the MSE and MDE calculation for time series of length N=1000, respectively. Here, since all values are defined in range of all scale, it is available to compare the entropy evaluation of the MDE and MSE. Both the MDE and MSE results have a smaller standard deviation at each scale than the results of length N=100 and 300. For the MSE result of Figure 4e, as the scale becomes larger than scale 5, the entropy values of chaotic series and white noise gradually decrease and are comparable on large scales. Compared to Figure 4c, it results in reduced standard deviation at each scale. 

The MDE results in Figure 4f indicate similar behaviors in the results in Figure 4d with the decreased standard deviation. Notably, the results of Figure 4b,d,f show that the MDE method leads to similar results in the complexity analysis for short-term and long-term time series, implying its insensitivity to the length of time series. In addition, MDE shows the smaller standard deviation than that of MSE and defined over all scales, indicating its superior stability and reliability over MSE.

### 3.2. Experiment Results Using Real Data

#### 3.2.1. ECG Dataset I

**Figure 5:**
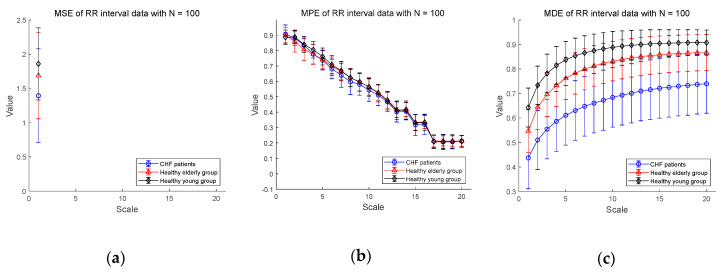

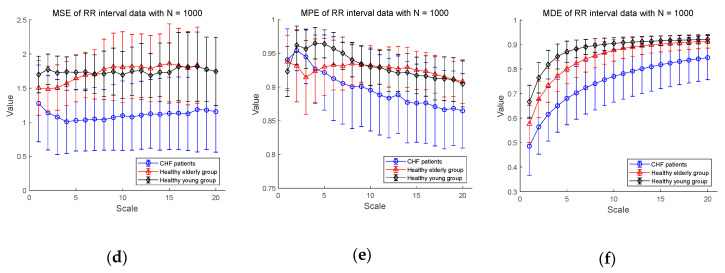
MSE, multiscale permutation entropy (MPE), and MDE results using real data (RR interval) for CHF patients, healthy elderly and healthy young groups: (**a**) MSE for N=100; (**b**) MPE for N=100; (**c**) MDE for N=100; (**d**) MSE for N=1000; (**e**) MPE for N=1000; (**f**) MDE for N=1000. Scale range of 1–20 were used and the value at each scale represents a mean ± standard deviation.

We show the experimental results using RR interval time series extracted from the ECG signal database measured for CHF patients, healthy elderly and healthy young groups in Figure 5. Figure 5a–c shows the MSE, MPE, and MDE results for time series of length N=100, respectively. In Figure 5a, the MSE values are not defined on most part of scales, indicating the shortcoming of MSE in analyzing a short-term RR interval time series. As for the results of MPE analysis in Figure 5b, the MPE values are present at all scales, but the entropy values decrease since the length of the time series get shorter as the multiscale process progresses. The distinction between CHF patients and healthy subjects seems to be difficult. On the other hand, the results of the MDE analysis in Figure 5c shows that the entropy values are defined over all scales for RR interval time series of length N=100. In addition, MDE is capable of reflecting the difference of the complexity of RR interval time series not only between CHF patients and healthy subjects, but also between healthy subject groups, i.e., between the elderly and the young groups. As the scale increases, the MDE values of three groups get higher. 

Next, Figure 5d–f shows the MSE, MPE, and MDE results for RR interval time series of length N=1000, respectively. In Figure 5d, in a situation where the length of the time series is long enough, the result of the MSE values are defined over most scales except for healthy elderly group on scales 19 and 20. The distinction between CHF patients and healthy subject groups appears to be available over most scales. However, the entropy values of the healthy young group are higher than those of the healthy elder one until the scale 7, but after that the distinction between the two groups is difficult. As for the results of MPE analysis in Figure 5e, the MPE values show a slight decrease, which is less than the result (N=100) in Figure 5b, and the distinction between the three groups seems possible. However, since the complexity of healthy subjects must be greater than the complexity of a CHF patients, it is possible to discriminate between CHF patients and healthy elderly group after the scale 5, and between CHF patients and healthy young group after the scale 2. In addition, the mean entropy values of the healthy young group are higher than those of the healthy elder group only on scales between 2 and 9, and the distinction is difficult on other scales. On the other hand, the MDE result in Figure 5f shows a similar evaluation results to Figure 5c in a situation where the length N=100. In addition, as the scale gets larger, the entropy values reached is larger than those for the short-term time series with reduced variance. The MDE behaviors shown in Figure 5 are closely consistent with the previous finding of the decreased complexity with aging and pathological status, whereas the MSE and MPE results do not agree with known behaviors of physiological complexity [6].

#### 3.2.2. ECG Dataset II

**Figure 6:**
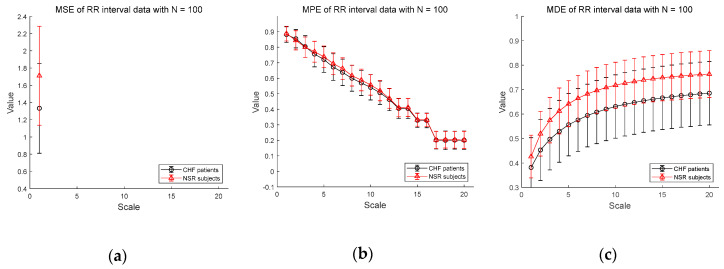

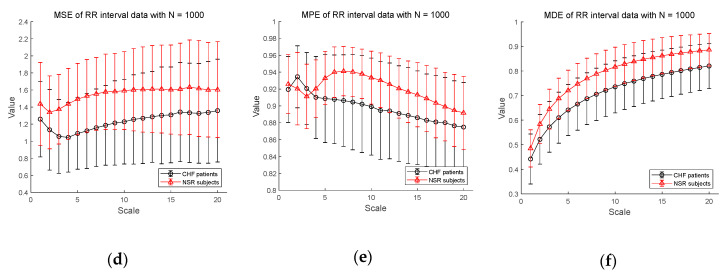
MSE, MPE, and MDE results using real data (RR interval) for other CHF patients and normal sinus rhythm (NSR) subjects: (**a**) MSE for N=100; (**b**) MPE for N=100; (**c**) MDE for N=100; (**d**) MSE for N=1000; (**e**) MPE for N=1000; (**f**) MDE for N=1000. Scale range of 1–20 were used and the value at each scale represents a mean ± standard deviation.

Figure 6 shows the results of entropy computation for RR interval time series obtained from CHF patients and NSR subject datasets to further evaluate the distinction performance between CHF patients and normal individuals. Figure 6a–c shows the MSE, MPE, and MDE results for time series of length N=100, respectively. MSE is not defined on most scales except scale 1 and MPE results show a dramatic decrease in entropy value as the scale becomes larger, indicating weak sensitivity of MPE on the length of a time series. Compared to conventional measures, MDE is able to reflect differences in the complexity of the RR interval time series between CHF patients and NSR group. Note that the MDE values increase as the scale gets higher, indicating robustness to the length of a time series. Next, in Figure 6d–f, the results of the MSE, MPE, and MDE are defined on most scales in situations where the length of the time series is long enough (N=1000). 

#### 3.2.3. Statistical Analysis for CHF Patients, Healthy Elderly, and Healthy Young Groups

To verify the distinction capability of entropy results between CHF patients, healthy elderly, and healthy young groups in Dataset I, statistical analysis was conducted. First, the Kolmogorov–Smirnov test is used to check whether the MDE and MSE results satisfy the normal distribution. If they follow a normal distribution, the *t*-test method was conducted to test the statistical difference between three datasets, and if not, the Mann–Whitney *U* test method was performed. Generally, if the *p*-value is less than 0.05, statistical significance is accepted. The analysis results are shown in Table 1, Table 2 and Table 3.

Table 1 shows the *p*-value of the MSE comparison result of RR interval time series when the length N is 100, 300, and 1000, respectively. As can be seen, for N=100 and 300, *p*-value computation is not available over most scales. For N=1000, the distinction using MSE values between CHF patients and healthy elderly group in scales from 1 to 18 and CHF patients and healthy young group over all scales are statistically significant. However, it fails to differentiate healthy elderly group from healthy young one over most scales (*p*-value > 0.05). The shadows represent the cases which the distinction is statistically insignificant.

In Table 2, the MPE results show that the shorter the length of the time series (N=100), the more difficult it is to distinguish between subject groups. As the length of RR interval increases, the discrimination performance gets better. However, even in N=1000, the distinction between groups fails in several cases. In addition, similar to the MSE results, the distinction between CHF patients and healthy subjects for N=1000 is statistically significant at most scales, but the distinction between healthy young and healthy elderly groups is available in limited scales. 

The statistical results of MDE in Table 3 exhibit that the distinctions using the MDE values between three groups are statistically significant for all three lengths of RR interval, i.e., N=100, 300, and 1000. Compared to the results of MSE and MPE, the MDE is capable of discriminating the complexities of different physiological groups for all scale values, implying its superiority over the conventional methods and insensitivity to the length of a time series.

## 4. Conclusions

We have presented an improved multiscale entropy, named MDE, for capturing the complexity of short-term HRV time series. For analysis of short-term HRV, the conventional MSE method suffers from unreliable computation due to its dependency to the length of time series. On the other hand, the proposed MDE method is capable of quantifying the complexity even for short-term time series by integrating DistEn with the moving-averaging process. The use of DistEn and moving-averaging multiscale approach leads to improved stability and reliability of entropy evaluation for short-term time series. Thus, the proposed MDE outperforms the conventional MSE and MPE in the sense that MDE is insensitive to the length of time series. Through synthetic data analysis, the proposed MDE is shown to be effective for describing the complexity of various time series such as chaotic series, white noise, MIX process, and periodic series. The experimental results using real ECG recordings from CHF patients and healthy subjects indicate that MDE is useful to reflect the degree of the decreased complexity of HRV accompanied by aging and disease. Through this work, the proposed MDE has shown its potential to be a promising solution for short-term HRV analysis.

## Figures and Tables

**Table 1:** Statistical analysis results of MSE for RR interval of the CHF patients, healthy young, and healthy elder groups. The shadows mean that the distinction between the groups is unclear. *C*, *E*, and *Y* represent CHF, Elder, and Young, respectively. *s* denotes scale factor and N/A denotes ‘Not Available’.

	MSE Statistical Results for RR Interval Time Series								
*s*	*p*-Value								
	N=100			N=300			N=1000		
	*C - E*	*C - Y*	*E - Y*	*C - E*	*C - Y*	*E - Y*	*C - E*	*C - Y*	*E - Y*
1	0.098	1.1 × 10^−6^	8.7 × 10^−5^	0.0014	2.5 × 10^−8^	3.6 × 10^−4^	1.3 × 10^−5^	4.8 × 10^−14^	8.5 × 10^−7^
2	N/A	N/A	N/A	4.7 × 10^−7^	5.5 × 10^−13^	1.0 × 10^−5^	6.7 × 10^−10^	1.5 × 10^−21^	8.0 × 10^−15^
3	N/A	N/A	N/A	2.4 × 10^−10^	1.8 × 10^−17^	1.9 × 10^−4^	8.4 × 10^−14^	3.2 × 10^−25^	5.3 × 10^−10^
4	N/A	N/A	N/A	3.9 × 10^−14^	3.1 × 10^−19^	0.019	2.3 × 10^−17^	2.2 × 10^−26^	2.7 × 10^−07^
5	N/A	N/A	N/A	1.3 × 10^−11^	2.8 × 10^−15^	0.311	1.3 × 10^−20^	1.9 × 10^−28^	0.005
6	N/A	N/A	N/A	N/A	N/A	N/A	1.9 × 10^−21^	8.8 × 10^−27^	0.089
7	N/A	N/A	N/A	N/A	N/A	N/A	3.7 × 10^−19^	1.2 × 10^−22^	0.512
8	N/A	N/A	N/A	N/A	N/A	N/A	1.6 × 10^−20^	1.1 × 10^−22^	0.847
9	N/A	N/A	N/A	N/A	N/A	N/A	3.5 × 10^−21^	2.2 × 10^−23^	0.353
10	N/A	N/A	N/A	N/A	N/A	N/A	1.4 × 10^−19^	5.1 × 10^−20^	0.175
11	N/A	N/A	N/A	N/A	N/A	N/A	5.2 × 10^−18^	6.8 × 10^−20^	0.673
12	N/A	N/A	N/A	N/A	N/A	N/A	1.6 × 10^−19^	2.3 × 10^−21^	0.359
13	N/A	N/A	N/A	N/A	N/A	N/A	1.3 × 10^−17^	1.0 × 10^−17^	0.086
14	N/A	N/A	N/A	N/A	N/A	N/A	3.7 × 10^−18^	5.0 × 10^−20^	0.853
15	N/A	N/A	N/A	N/A	N/A	N/A	1.9 × 10^−13^	2.2 × 10^−14^	0.448
16	N/A	N/A	N/A	N/A	N/A	N/A	1.6 × 10^−13^	4.7 × 10^−16^	0.945
17	N/A	N/A	N/A	N/A	N/A	N/A	1.0 × 10^−11^	1.6 × 10^−14^	0.695
18	N/A	N/A	N/A	N/A	N/A	N/A	1.6 × 10^−15^	2.2 × 10^−19^	0.936
19	N/A	N/A	N/A	N/A	N/A	N/A	N/A	3.2 ×10^−16^	N/A
20	N/A	N/A	N/A	N/A	N/A	N/A	N/A	4.1 × 10^−13^	N/A

**Table 2:** Statistical analysis results of MPE for RR interval of the CHF patients, healthy young, and healthy elder groups. The shadows mean that the distinction between the groups is unclear. *C*, *E*, and *Y* represent CHF, Elder, and Young, respectively. *s* denotes scale factor.

	MPE Statistical Results for RR Interval Time Series								
*s*	*p*-Value								
	N=100			N=300			N=1000		
	*C - E*	*C - Y*	*E - Y*	*C - E*	*C - Y*	*E - Y*	*C - E*	*C - Y*	*E - Y*
1	0.389	6.6 × 10^−7^	6.5 × 10^−5^	0.255	1.2 × 10^−7^	8.9 × 10^−6^	0.157	8.6 × 10^−8^	5.6 × 10^−4^
2	0.018	0.101	5.8 × 10^−5^	0.006	0.006	1.1 × 10^−7^	0.005	4.1 × 10^−4^	7.9 × 10^−7^
3	3.0 × 10^−4^	0.519	2.9 × 10^−4^	4.6 × 10^−6^	0.005	1.6 × 10^−14^	9.2 × 10^−9^	0.012	5.9 × 10^−13^
4	0.363	1.9 × 10−^5^	1.8 × 10^−4^	0.674	8.6 × 10^−13^	1.5 × 10^−16^	0.030	5.2 × 10^−12^	4.8 × 10^−19^
5	0.139	6.3 × 10^−8^	0.001	0.935	1.6 × 10^−9^	7.3 × 10^−13^	0.776	1.8 × 10^−15^	1.2 × 10^−16^
6	0.059	0.004	0.241	0.033	4.2 × 10^−9^	1.1 × 10^−5^	0.017	7.5 × 10^−16^	9.5 × 10^−10^
7	0.102	1.2 × 10^−8^	0.655	0.002	3.7 × 10^−7^	0.007	0.007	8.8 × 10^−13^	3.2 × 10^−6^
8	0.292	5.8 × 10^−7^	0.619	0.004	4.9 × 10^−4^	0.428	1.1 × 10^−5^	3.5 × 10^−8^	0.233
9	0.450	4.3 × 10^−6^	0.129	9.7 × 10^−4^	1.6 × 10^−4^	0.567	5.3 × 10^−5^	1.6 × 10^−5^	0.720
10	0.433	4.3 × 10^−4^	0.454	6.3 × 10^−4^	7.0 × 10^−6^	0.193	1.4 × 10^−6^	1.4 × 10^−5^	0.387
11	0.104	1.7 × 10^−5^	0.965	2.8 × 10^−5^	9.0 × 10^−5^	0.656	1.8 × 10^−8^	4.3 × 10^−8^	0.701
12	0.034	0.050	0.873	1.6 × 10^−4^	7.2 × 10^−8^	0.041	8.6 × 10^−10^	1.7 × 10^−7^	0.159
13	0.025	0.062	0.686	9.1 × 10^−7^	7.9 × 10^−6^	0.701	9.6 × 10^−9^	1.7 × 10^−5^	0.009
14	0.702	0.914	0.780	1.2 × 10^−7^	1.5 × 10^−4^	0.056	4.8 × 10^−13^	5.8 × 10^−9^	0.020
15	0.912	0.779	0.854	3.7 × 10^−5^	3.0 × 10^−4^	0.505	3.4 × 10^−11^	2.9 × 10^−7^	0.020
16	0.074	0.411	0.296	1.1 × 10^−4^	2.7 × 10^−4^	0.745	2.6 × 10^−13^	5.9 × 10^−10^	0.070
17	0.821	0.520	0.648	3.8 × 10^−5^	2.3 × 10^−4^	0.672	5.4 × 10^−10^	6.1 × 10^−8^	0.309
18	0.171	0.531	0.431	1.1 × 10^−5^	5.9 × 10^−6^	0.880	2.6 × 10^−9^	1.4 × 10^−8^	0.729
19	0.448	0.437	0.103	3.7 × 10^−5^	1.4 × 10^−1^	0.758	2.0 × 10^−11^	6.1 × 10^−11^	0.947
20	0.272	0.556	0.588	0.002	0.005	0.671	1.0 × 10^−9^	3.9 × 10^−8^	0.496

**Table 3:** Statistical analysis results of MDE for RR interval of the CHF patients, healthy young, and healthy elder groups. *C*, *E*, and *Y* represent CHF, Elder, and Young, respectively. *s* denotes scale factor.

	MDE Statistical Results for RR Interval Time Series								
*s*	*p*-Value								
	N=100			N=300			N=1000		
	*C - E*	*C - Y*	*E - Y*	*C - E*	*C - Y*	*E - Y*	*C - E*	*C - Y*	*E - Y*
1	2.8 × 10^−15^	3.6 × 10^−26^	6.9 × 10^−15^	2.1 × 10^−11^	9.2 × 10^−26^	5.5 × 10^−16^	1.8 × 10^−10^	3.1 × 10^−26^	1.1 × 10^−16^
2	1.7 × 10^−18^	7.2 × 10^−31^	1.3 × 10^−13^	2.7 × 10^−15^	7.1 × 10^−32^	1.1 × 10^−14^	1.7 × 10^−14^	1.6 × 10^−31^	1.0 × 10^−16^
3	4.9 × 10^−21^	5.5 × 10^−33^	2.0 × 10^−12^	6.5 × 10^−18^	2.3 × 10^−34^	2.7 × 10^−14^	2.8 × 10^−17^	5.6 × 10^−34^	3.5 × 10^−17^
4	1.1 × 10^−21^	1.5 × 10^−33^	4.3 × 10^−12^	1.3 × 10^−18^	1.6 × 10^−34^	1.3 × 10^−14^	3.9 × 10^−35^	3.9 × 10^−35^	4.7 × 10^−18^
5	2.5 × 10^−23^	3.3 × 10^−34^	1.5 × 10^−11^	1.2 × 10^−13^	9.7 × 10^−35^	1.2 × 10^−14^	1.8 × 10^−35^	1.8 × 10^−35^	6.1 × 10^−17^
6	2.1 × 10^−23^	1.7 × 10^−34^	4.8 × 10^−11^	2.6 × 10^−20^	5.7 × 10^−35^	2.4 × 10^−14^	5.4 × 10^−35^	5.4 × 10^−35^	2.0 × 10^−15^
7	3.1 × 10^−23^	5.8 × 10^−34^	3.0 × 10^−10^	4.9 × 10^−20^	2.2 × 10^−34^	2.4 × 10^−13^	1.1 × 10^−33^	1.1 × 10^−33^	8.1 × 10^−13^
8	2.1 × 10^−23^	8.0 × 10^−34^	2.0 × 10^−9^	4.6 × 10^−20^	3.4 × 10^−34^	3.0 × 10^−12^	1.6 × 10^−32^	1.6 × 10^−32^	1.4 × 10^−10^
9	6.7 × 10^−23^	2.1 × 10^−33^	4.5 × 10^−9^	1.9 × 10^−20^	4.4 × 10^−34^	9.1 × 10^−11^	6.9 × 10^−32^	6.9 × 10^−32^	1.9 × 10^−9^
10	6.9 × 10^−23^	2.1 × 10^−33^	9.6 × 10^−9^	3.3 × 10^−20^	1.4 × 10^−33^	2.9 × 10^−9^	9.6 × 10^−31^	9.6 × 10^−31^	8.9 × 10^−8^
11	1.7 × 10^−22^	3.3 × 10^−33^	2.0 × 10^−8^	3.8 × 10^−20^	2.3 × 10^−33^	2.5 × 10^−8^	3.3 × 10^−30^	3.3 × 10^−30^	4.8 × 10^−6^
12	2.4 × 10^−22^	2.9 × 10^−33^	2.5 × 10^−8^	4.6 × 10^−20^	1.5 × 10^−32^	1.6 × 10^−7^	5.9 × 10^−29^	5.9 × 10^−29^	1.8 × 10^−5^
13	2.6 × 10^−22^	1.1 × 10^−32^	5.7 × 10^−8^	1.9 × 10^−20^	4.6 × 10^−32^	7.4 × 10^−7^	2.4 × 10^−27^	2.4 × 10^−27^	1.4 × 10^−4^
14	8.0 × 10^−22^	7.9 × 10^−33^	1.0 × 10^−7^	8.8 × 10^−20^	4.4 × 10^−31^	2.1 × 10^−6^	2.8 × 10^−25^	2.8 × 10^−25^	6.0 × 10^−4^
15	3.2 × 10^−21^	4.5 × 10^−32^	2.3 × 10^−7^	3.5 × 10^−19^	3.8 × 10^−30^	4.8 × 10^−6^	4.0 × 10^−23^	4.0 × 10^−23^	0.001
16	1.0 × 10^−20^	1.1 × 10^−31^	3.8 × 10^−7^	4.7 × 10^−18^	1.1 × 10^−28^	4.8 × 10^−6^	7.6 × 10^−21^	7.6 × 10^−21^	8.8 × 10^−4^
17	3.5 × 10^−20^	2.4 × 10^−31^	4.4 × 10^−7^	1.6 × 10^−17^	2.7 × 10^−27^	1.7 × 10^−5^	2.2 × 10^−18^	2.2 × 10^−18^	0.004
18	7.9 × 10^−20^	3.4 × 10^−31^	9.6 × 10^−7^	1.6 × 10^−17^	4.0 × 10^−27^	3.7 × 10^−5^	1.1 × 10^−16^	1.1 × 10^−16^	0.005
19	2.1 × 10^−19^	1.4 × 10^−30^	1.4 × 10^−6^	7.6 × 10^−17^	1.8 × 10^−26^	1.2 × 10^−9^	5.8 × 10^−16^	5.8 × 10^−16^	0.006
20	5.3 × 10^−19^	1.9 × 10^−30^	4.0 × 10^−6^	1.7 × 10^−16^	7.0 × 10^−26^	1.1 × 10^−5^	2.4 × 10^−14^	2.4 × 10^−14^	0.015
